# Ultrasensitive electrochemical detection of methyl parathion pesticide based on cationic water-soluble pillar[5]arene and reduced graphene nanocomposite†

**DOI:** 10.1039/c8ra08555b

**Published:** 2019-01-02

**Authors:** Xiaoping Tan, Yan Liu, Tingying Zhang, Shasha Luo, Xi Liu, Hexiang Tian, Yang Yang, Chunlian Chen

**Affiliations:** Key Lab of Inorganic Special Functional Materials, Chongqing Municipal Education Commission, School of Chemistry and Chemical Engineering, Yangtze Normal University Fuling 408100 China xptan@yznu.cn

## Abstract

We report a rapid, sensitive and selective electrochemical sensor based on pillar[5]arene (CP5) reduced graphene (rGO) nanohybrid-modified glassy carbon electrode CP5-rGO/GCE for the trace detection of methyl parathion (MP) by differential pulse voltammetry (DPV) for the first time. Compared to beta-cyclodextrin (β-CD)-functionalized reduced graphene (rGO)-modified GCE β-CD-rGO/GCE, the proposed CP5-rGO/GCE sensor exhibits excellent electrochemical catalytic activity, rapid response, high sensitivity, good reproducibility and anti-interference ability towards MP. The recognition mechanism of β-CD/MP and CP5/MP was studied by ^1^H NMR. The results indicate a higher supramolecular recognition capability between CP5 and MP compared to that between β-CD and MP. The β-CD-rGO and CP5-rGO nano-composites were prepared *via* a wet chemistry approach. The resulting nano-composites have been characterized by thermogravimetric analysis (TGA), fourier transform infrared spectrometry (FTIR), charge transfer resistance (*R*_ct_) and zeta potential. The CP5-rGO/GCE combines the merits of CP5 and rGO, and is used for quantitative detection of MP. It has a low detection limit of 0.0003 μM (S/N = 3) and a linear response range of 0.001–150 μM for MP. This method has been used to detect MP in soil and waste water samples with satisfactory results. This study provides a promising electrochemical sensing platform and is a promising tool for the rapid, facile and sensitive analysis of MP.

## Abstract 2

A promising electrochemical sensing platform for the detection of methyl parathion based on cationic water-soluble pillar[5]arene reduced graphene nanocomposite.

## Graphical abstract

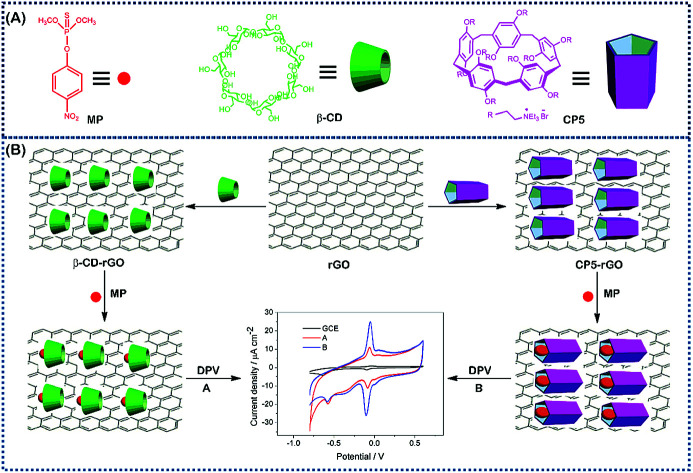

## Introduction

Organophosphorus pesticides (OPs) have high toxicity, which often lead to difficulty breathing and death.^1,2^ The broad use of OPs for pest control has caused serious concerns in regards to human health, food safety and environmental protection.^3,4^ Methyl parathion (MP) is a common OP used in fish hatcheries and agriculture.^5,6^ It is potentially toxic and may be harmful to human health.^7,8^ Therefore, the rapid and sensitive determination of MP is increasingly important.^9–11^ MP is currently determined by gas chromatography-mass spectrometry,^12^ immunoassays,^13^ molecular imprinting,^14^ liquid chromatography-mass spectrometry and mass spectrometry.^15,16^ However, these methods often need complicated sample pretreatments or suffer from high costs. Electroanalytical methods are simple, rapid and cheap, and have been used for the determination of MP and other pesticides.^17–20^

For example, Tonle *et al.* prepared a Gemini surfactant-intercalated clay-modified electrode for the determination of MP with a linear dynamic range of 4.0 × 10^−7^ to 1.0 × 10^−6^ M.^21^ Xu *et al.* employed a poly (malachite green)/graphene nanosheet–Nafion (PMG/GNs–NF) composite film modified glassy carbon electrode to detect MP with a linear range of 2 × 10^−8^ to 1.5 × 10^−6^ M and a detection limit of 2 × 10^−9^ M.^22^ Dai *et al.* used Nafion as an additive, and drop-casted ZrO_2_-CNFs onto a glassy carbon electrode (ZrO_2_-CNF/GCE). The modified electrode was then applied to detect MP with a linear range of 1 × 10^−9^ to 2 × 10^−8^ M and a detection limit of 3.4 × 10^−10^ M.^23^ Although these electroanalytical methods were rapid and sensitive, some of them exhibited poor selectivity. Therefore, tools that are both selective and sensitive to detect MP remain an unmet need.

Macrocyclic arenes play an important role in the field of supramolecular chemistry.^24–27^ They contain aromatic rings linked by methylenes or heteroatoms, such as oxygen, nitrogen and sulfur.^28^ Conventional macrocyclic arenes, such as cyclodextrins, calixarenes, calixpyrroles, resorcinarenes and cyclotriveratrylenes, with different electronic constitutions and topological structures, exhibit a variety of host–guest properties.^29,30^ Cyclodextrins (CDs) are toroidal in shape with a hydrophilic exterior and a hydrophobic inner cavity. These interesting characteristics enable them to selectively bind various guest molecules and form stable host/guest complexes in their hydrophobic cavity.^31,32^

Pillar[*n*]arenes were first reported by Ogoshi *et al.* in 2008.^33^ Pillar[*n*]arenes are composed of *p*-hydroquinone units held together by methylene bridges linking the *para*-positions of the *p*-hydroquinone units in cyclic arrays. Currently, pillar[*n*]arenes are recognized as important players in supramolecular chemistry due to their easy synthesis, versatile functionality, unique pillar shaped structure, excellent host–guest properties and inartificial supramolecular assembly characteristics, and this has resulted in numerous electrochemical and biomedical material applications.^34–38^

In 2015, Li *et al.* first reported a novel coumarin-pillar[5]arene (P5C10) as a selective fluorescent probe for MP, however it suffered from limited selectivity and sensitivity. Thus, they constructed an electrochemical sensor based on pillar[5]arene for the determination of MP.^39^ The use of a water-soluble macrocyclic host that interacts with graphene *via* π–π stacking and hydrogen bonding interactions can improve selectivity and sensitivity.^40–44^ Graphene has unique mechanical, thermal, and electronic properties as well as a high surface area, low cost, and low toxicity arising from its strict 2D structure.^45,46^ It has many applications in sensors, drug carriers and other technological fields. Mao *et al.* reported a sensor for detecting paraquat in living cells based on pillar[5]arene and graphene.^47^ Zhou *et al.* reported an electrochemical sensing platform based on reduced graphene and amphiphilic pillar[5]arene-gold nanoparticles.^48^ Zhao *et al.* reported a competitive fluorescence sensing for acetaminophen.^49^ Therefore, the composites of pillar[*n*]arene and graphene demonstrate some special performances.

**Fig. 1:**
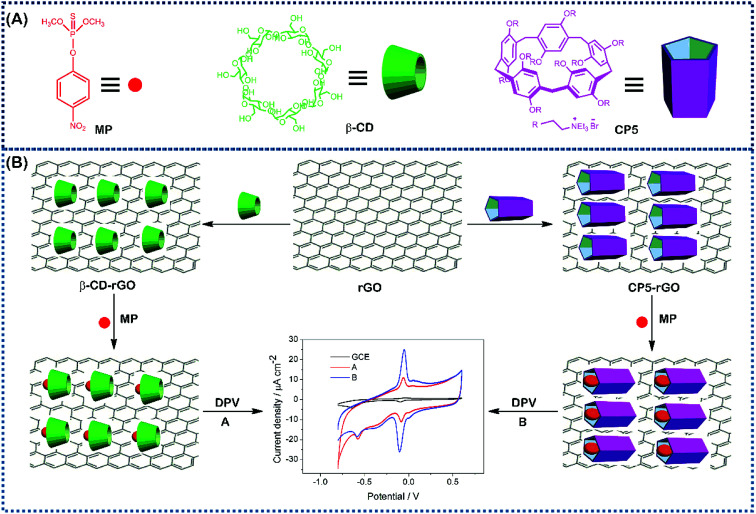
The corresponding cartoon representations of MP, CP5 and β-CD and the illustration of CP5-rGO (A) and β-CD-rGO (B) nanohybrids-based electrochemical sensing method towards methyl parathion.

Herein, we describe a rapid and sensitive electrochemical sensor based on cationic pillar[5]arene (CP5)-reduced graphene (rGO) nanohybrid material-modified glassy carbon electrode (CP5-rGO/GCE). This material was successfully fabricated for the trace determination of MP by DPV for the first time. Compared to beta-cyclodextrin-functionalized reduced graphene composite (β-CD-RGO), the electrochemical sensor has higher electrical signal and recognition capability. This is due to the higher recognition of CP5 compared to that of β-CD towards MP. The electrochemical sensing platform based on CP5-rGO/GCE is illustrated in Fig. 1. This method is simple, highly sensitive and selective, and has been successfully applied to MP detection in soil and tap water samples. The proposed approach can also be extended to other potential applications in environmental, industrial, and biological samples, such as drug metabolites in organisms.

## Materials and methods

### Chemicals and materials

**Scheme 1:**
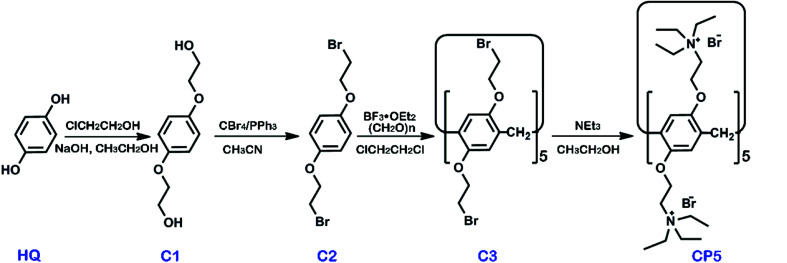
The synthesis of cationic water-soluble pillar[5]arene CP5.

β-Cyclodextrin (β-CD, >99%) and graphene oxide (GO) were obtained from Shanghai TITAN Tech Co., Ltd. (Shanghai, China). Cationic water-soluble pillar[5]arene (CP5) was synthesized *via* the previously published procedures and the synthetic route is shown in Scheme 1 with synthetic details described in the ESI.† Other chemicals utilized were of analytical grade and were used without further purification. All of the aqueous solutions were prepared *via* deionized water (DW, 18 MΩ cm). The apparatus and other reagents are shown in the SI. The structure and purity of all compounds were confirmed by ^1^H NMR and ^13^C NMR (see Fig. S1–S8†).

### Synthesis of CP5-rGO and β-CD-rGO composites

CP5 (20 mg) and GO (20 mg) were dissolved in deionized water (50 mg). Ammonia (3 mL) and ascorbic acid (200 mg) were then added, and the reaction mixture was stirred for 20 h at room temperature. After the reaction was completed, the CP5-rGO composite was obtained after being centrifuged at 12 000 rpm for 40 min and was washed 4 times. The CP5-rGO powder was obtained by freeze drying for further characterization. The preparation of the β-CD-rGO composite was similar to that of the CP5-rGO composite.

### Fabrication of the modified electrodes

A glassy carbon electrode (GCE, 3 mm in diameter) was sequentially polished with Al_2_O_3_ powder (0.3 and 0.05 mm) and then sonicated in ethanol and DW to remove the physically adsorbed substances and dried in air. The CP5-rGO nanohybrid material was dissolved in DW at a concentration of 0.5 mg mL^−1^ with ultrasonic treatment for 20 min. This resulted in a homogeneous suspension. β-CD-rGO (0.5 mg mL^−1^) or rGO homogeneous suspensions were also similarly obtained. To prepare the CP5-rGO-modified electrode, 5 μL of the CP5-rGO suspension was dropped onto the electrode surface and dried in air. The resulting electrode was termed as CP5-rGO/GCE. β-CD-rGO/GCE and rGO/GCE were also prepared *via* the similar procedure.

### Electrochemical measurements

Cyclic voltammetry (CV) was achieved in phosphate buffer (0.1 M pH 6.0) containing 20 μM of MP. Differential pulse voltammetry (DPV) was realized in phosphate buffer (0.1 M pH 6.0) from 0.4 to −0.4 V with a pulse amplitude of 0.05 V and a pulse width of 0.05 s containing different concentrations of MP. Electrochemical impedance spectroscopy (EIS) was recorded in the frequency range of 10^−1^ to 10^5^ Hz with an amplitude of 5 mV employing [Fe(CN)_6_]^3−/4−^ (2.0 mM) redox couple (1 : 1) with KCl (0.1 M) as the supporting electrolyte. All of the electrochemical measurements were carried out at room temperature.

## Results and discussion

### Characterization of the β-CD-rGO and CP5-rGO composites

**Fig. 2:**
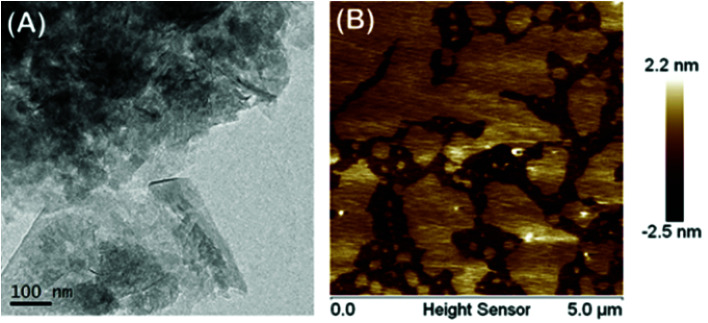
TEM (A) and AFM (B) of CP5-rGO nano-material.

GO and CP5-rGO were characterized by the UV-vis spectroscopy and the results are shown in Fig. S9.† The absorption peak of graphene oxide dispersion at 230 nm gradually redshifts to 260 nm, which indicates that the reduction process between graphene oxide and reduced graphene successfully proceeds under the alkaline conditions. We further characterized the microstructure of CP5-rGO by TEM (Fig. 2A). The TEM image reveals that CP5-rGO material is randomly aggregated thin, wrinkled sheets closely associated with each other. As shown in Fig. 2B, the AFM morphology of CP5-rGO is a nano-sheet structure, indicating the single-layer feature of graphene. It was difficult to distinguish the CP5 molecules in the TEM image, and thus PP5-rGO composite was further characterized by FTIR spectroscopy, zeta potential measurements, TGA and XPS analysis.

**Fig. 3:**
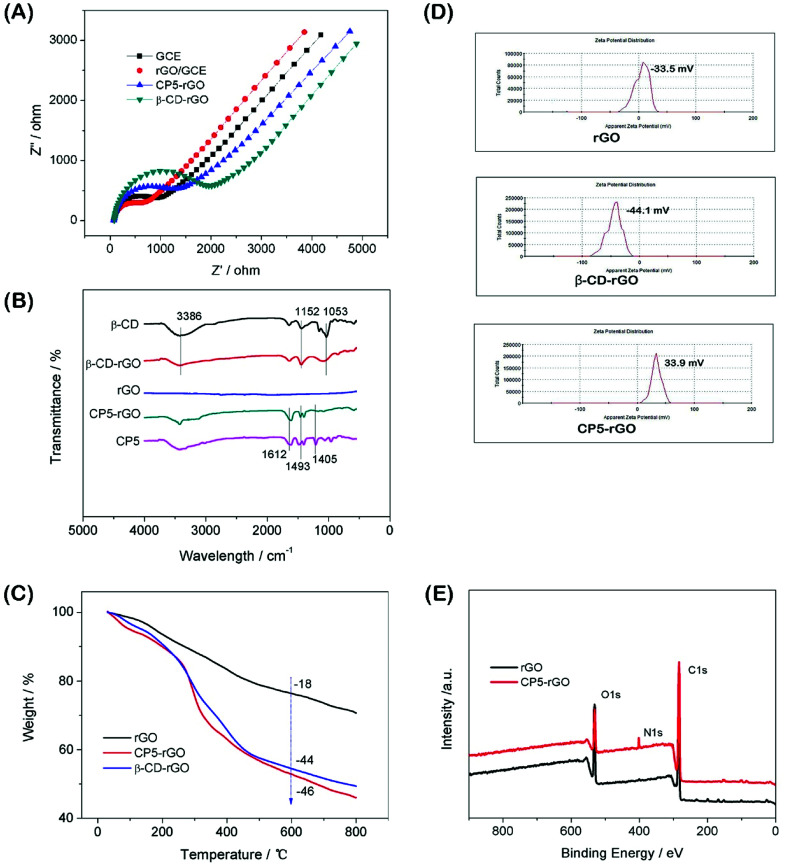
The EIS characterization of GCE, β-CD-rGO/GCE and CP5-rGO/GCE *via* [Fe(CN)_6_]^3−/4−^ (2.0 mM) redox couple (1 : 1) with KCl (0.1 M) as supporting electrolyte (A). FTIR spectra of β-CD, β-CD-rGO, rGO, CP5-rGO and CP5 (B). TGA curves of rGO, CP5-rGO and β-CD-rGO (C). Zeta potentials of rGO, β-CD-rGO and CP5-rGO (D). XPS survey spectra of rGO and CP5-rGO (E).

Electrochemical characterization of the modified electrodes used a value of charge transfer resistance (*R*_ct_) with a potential of 0.1 V. The frequency ranges are 10^−1^ to 10^5^ Hz using 2.0 mM [Fe(CN)_6_]^3−/4−^ redox couple (1 : 1) with 0.1 M KCl as the supporting electrolyte.^50^ The value of the electron-transfer resistance (*R*_ct_) of the modified electrode is estimated by the semicircle diameter. Fig. 3A illustrates the EIS of the bare GCE, rGO/GCE, CP5-rGO/GCE and β-CD-rGO/GCE. Evidently, the bare GCE exhibits a semicircle portion, and the value of *R*_ct_ is estimated to be 900 Ω. The rGO/GCE is estimated to be 750 Ω, which attributes to high conductivity of rGO. When the CP5-rGO and β-CD-rGO modified the bare GCE, the semicircles were increased to 1500 Ω and 2100 Ω, respectively. This is caused by the organic macromolecules of CP5 and β-CD hindering the electron transfer. This renders the interfacial charge transfer difficult. The results show that β-CD and CP5 molecules are successfully immobilized on the surface of rGO. The *R*_ct_ value of β-CD-rGO/GCE is higher than that of CP5-rGO/GCE indicating that the electrical conductivity of β-CD is worse than CP5 due to the quaternary ammonium bromide of CP5.

Fourier transform infrared spectroscopy (FTIR) results of the β-CD-rGO and CP5-rGO composites are observed in Fig. 3B. Fig. 3B shows no significant absorption in the FTIR spectrum of the reduced graphene (rGO) indicating that the reduction effect is very good. The characteristic absorption peak of β-CD appears in the FTIR spectrum of the β-CD-rGO composite. The stretching vibration of C–O is 1053 cm^−1^ and the asymmetric stretching vibration of –COC– is 1152 cm^−1^. The stretching vibration corresponding to the O–H group appears at 3386 cm^−1^. The presence of these characteristic peaks indicates that the composite β-CD-rGO has been successfully prepared. Similarly, the characteristic absorption peak of CP5 can be observed in the FTIR spectrum of the composite CP5-rGO. The most significant characteristic peak on CP5 is the vibration absorption bands of the benzene ring at 1612 cm^−1^, 1493 cm^−1^ and 1405 cm^−1^. These peaks show that CP5 has been successfully compounded by rGO.

The resulting β-CD-rGO, CP5-rGO and the other related materials are also characterized by thermogravimetry (TGA) as shown in Fig. 3C. The pristine rGO has a minor loss in mass (18%) at about 600 °C because of the pyrolysis of a very small amount of the labile oxygen-containing functional groups. The β-CD-rGO composite shows an abrupt mass loss when the temperature is approximately 270 °C due to the decomposition of β-CD; the mass loss reached about 44 wt% when the temperature is 600 °C. Meanwhile, the CP5-rGO composite also exhibits an abrupt mass loss at about 270 °C due to the decomposition of CP5; the mass loss reached about 46 wt% when the temperature is approximately 600 °C, indicating that the β-CD-rGO and CP5-rGO nanohybrids are successfully fabricated.

The average zeta potentials of rGO, β-CD-rGO and CP5-rGO are −23.3, −44.1 and 34.4 mV, respectively, as shown in Fig. 3D. Compared to the zeta potential of rGO, the zeta potential of CP5-rGO increases by approximately 57.7 mV, and this is ascribed to the positive charge of –N(C_2_H_5_)_3_^3+^ in the CP5. However, compared to the zeta potential of rGO, the zeta potential of β-CD-rGO decreases by approximately 20 mV because of the negative charge on the β-CD. Generally, the zeta potentials of β-CD-rGO and CP5-rGO are higher than 30 mV suggesting that β-CD-rGO and CP5-rGO dispersions are colloidally stable. The zeta potential of rGO is −23.3 mV (lower than 30 mV), indicating the poor aqueous dispersion of rGO. These results are in accordance with the photographs of r-GO, β-CD-rGO and CP5-rGO aqueous dispersions, as shown in Fig. S10.†

As shown in Fig. 3E, X-ray photoelectron spectroscopy (XPS) analysis is used to confirm the electronic structure and compositions of rGO and CP5-rGO. Fig. 3E shows a significant N 1s peak for the resulting CP5-rGO attributed to the CP5; no N signal is detected on the rGO film, which further indicates the successful chemical modification of CP5 on rGO. Therefore, these results suggest that the CP5 or β-CD has been successfully grafted on the surface of rGO.

### Optimization of experimental conditions for the detection of MP at CP5-rGO/GCE

The electrochemical control experiments are performed to optimize MP detection. The accumulation time has significant effects on the performance of the sensor. The pH value of PBS buffer, accumulation potential and accumulation time were studied for their effects on the DPV peak currents of 10 μM MP at the CP5-rGO modified electrode in 0.1 M PBS.

The pH of the PBS buffer is a key factor underlying the electrochemical signal of the MP. It is performed for different pH values (4 to 8) with 10 μM MP at the CP5-rGO/GCE (Fig. S11(A)†). The peak current gradually enhances with increasing the pH and reaches a maximum value at pH 6.0. This may be due to the degradation of OP compounds and the reduced process of MP under H^+^ condition.^51^ Fig. S11(B)† shows that the corresponding DPV peak current value gradually enhances with increasing the accumulation potential and reaches a maximum value at −0.1 V, which suggests that the reduction of MP at CP5-rGO/GCE is maximized. As shown in Fig. S11(C),† the DPV peak current value reaches a maximum at 150 s of accumulation time, indicating that the reduction of MP at CP5-rGO/GCE is saturated. Therefore, 0.1 M pH 6.0 PBS, −0.1 V accumulation potential, and 170 s accumulation time are used for the following experiments.

### Electrochemical behavior of MP in different modified electrodes

**Fig. 4:**
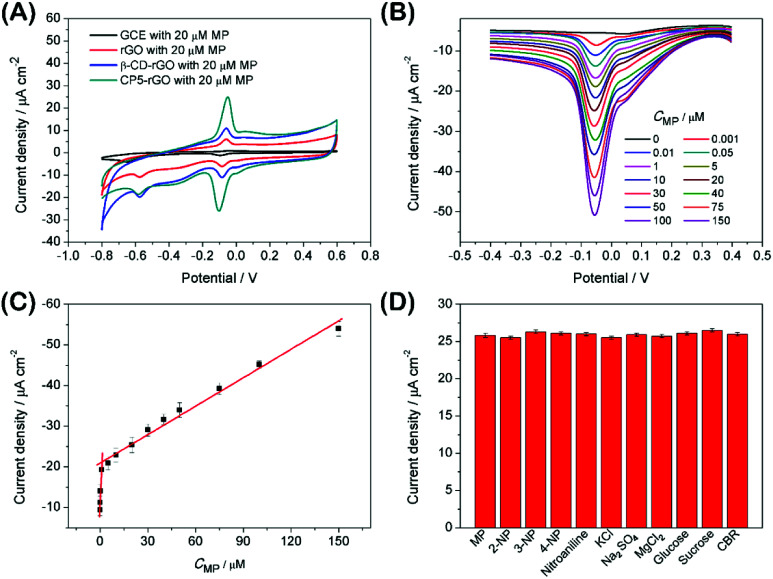
The CV curves obtained for the redox reaction of 20 μM MP in PBS (0.1 M, pH 6.0) at bare GCE, rGO/GCE, β-CD-rGO/GCE and CP5-rGO/GCE (A). DPV curves obtained for the reduction of MP at CP5-rGO/GCE for different concentrations (B). Calibration plots of the reduction peak current at CP5-rGO/GCE *versus* concentration of MP under optimal conditions (C). Selectivity studies with different species in the developed MP detection strategy use DPV and keep all of the parameters constant. The MP concentration is 20 μM against the concentration of all of the other substances—this is held at 1.0 mM (D).

The electrochemical behavior of MP has been investigated using a variety of modified electrodes. The standard MP was diluted to 50 μM as a stock solution in PBS (0.1 M, pH 6.0) buffer solution, and the modified electrode GCE, rGO/GCE, β-CD-rGO/GCE and CP5-rGO/GCE are measured for CV in a 20 μM MP solution (Fig. 4A). Fig. 4A shows that the bare GCE has two weak reduction peaks. Compared to the bare GCE, the peak current value of MP is enhanced at rGO/GCE. This enhancement indicates that the high surface area and high conductivity of the rGO promotes the effective electrode area and increases the catalytic activity towards MP oxidation and reduction. With respect to β-CD-rGO/GCE, the redox currents of MP are improved in comparison with rGO-modified GCE indicating that the β-CD molecules with excellent supramolecular recognition capability form inclusion complexes with MP. However, the redox current increases remarkably when the CP5-rGO nanohybrid is immobilized onto the surface of a GCE. This might be because the CP5 molecules have a large cavity, and the cavity has stronger supramolecular recognition capability than β-CD.

**Fig. 5:**
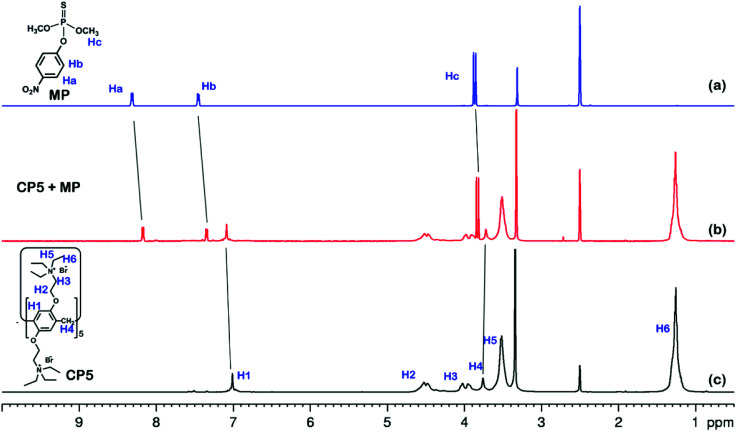
^1^H NMR spectra (500 MHz, DMSO-*d*_6_, 298 K): (a) MP (10.00 mM); (b) a mixture of 10.00 mM MP and 10.00 mM CP5; (c) CP5 (10.00 mM).

**Fig. 6:**
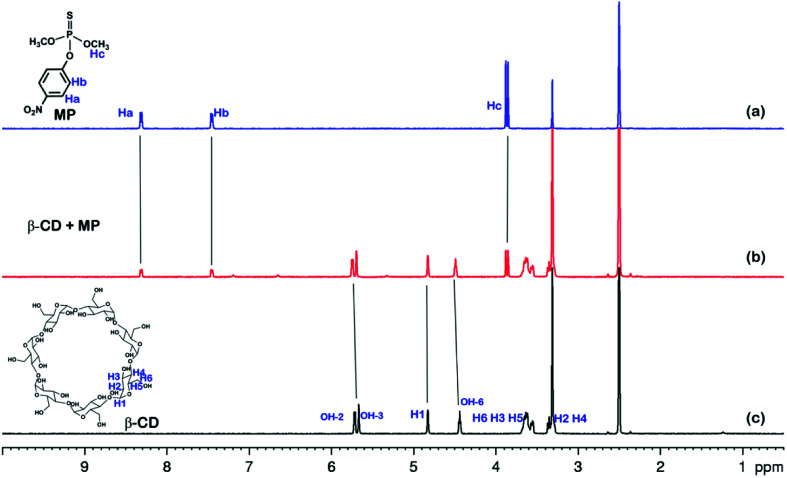
^1^H NMR spectra (500 MHz, DMSO-*d*_6_, 298 K): (a) MP (10.00 mM); (b) a mixture of 10.00 mM MP and 10.00 mM β-CD; (c) β-CD (10.00 mM).

The ^1^H NMR spectra of the host-guest complexation between CP5 and MP, and β-CD and MP are investigated in DMSO-*d*_6_ to illustrate the recognition mechanism of β-CD/MP and CP5/MP. As shown in Fig. 5, the signals of the protons (Ha and Hb) of MP have significantly shifted upfield, and the protons (H1) from CP5 have significantly shifted downfield. These indicated host-guest complexation by the hydrophobic interactions, π–π interactions and deficient-rich electron interactions between CP5 and MP.^39,51–54^ However, the proton signals (Ha and Hb) of MP did not shift. The hydroxyl protons of β-CD shifted slightly downfield (Fig. 6). This phenomenon may be caused by the hydrogen bond interaction between β-CD and MP. Thus, the CP5 exhibits higher recognition capability towards MP than β-CD. There is higher electrical signal with CP5-rGO modified GCE.

### The mechanism of MP on the modified electrode

**Scheme 2:**
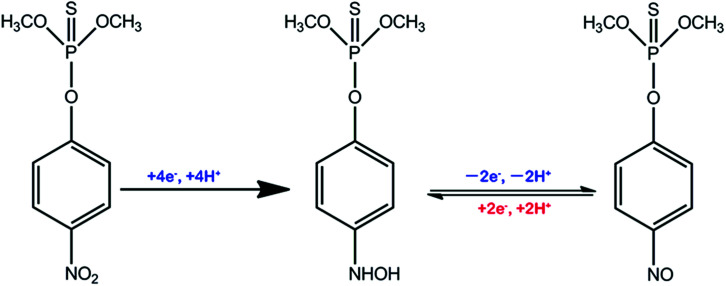
The chemical reaction mechanism of MP in CP5-rGO-modified electrode.

The reaction mechanism of MP has been extensively researched.^55–58^Scheme 2 contributes to a sharp irreversible reduction peak at around −0.58 V that corresponds to the reduction of the nitro group (–NO_2_) to the hydroxylamine moieties (–NHOH) by a four-electron reduction process. Then, during the anodic scan, the hydroxylamine moieties are reversibly oxidized to the nitroso group (–NO) with an oxidation peak (around 0 V). The nitroso materials are reversibly reduced to the hydroxylamine group and have another reduction peak at around −0.1 V. This pair of reversible redox peaks is ascribed to a two-electron transfer redox process.

### Analytical performance, selectivity and real sample analysis

The analytical performance was examined with DPV measurements to study potential applications of the CP5-rGO-modified GCE. Fig. 4B shows the DPV curves of MP on CP5-rGO/GCE. The peak current increases with the increasing MP concentrations. The corresponding calibration curves for MP can also be obtained (Fig. 4C). This method can achieve a low detection limit of 0.0003 μM (S/N = 3) and a linear response range of 0.001–150 μM. The corresponding regression equations are *I* (μA) = 8.027*C* (μM) + 11.33 and *I* (μA) = 0.233*C* (μM) + 20.918, and the correlation coefficients are 0.973 and 0.985, respectively. As shown in Table S1,† although some of the reported studies have lower detection limits than our study, our study exhibits wider linear range and the detection limit is a little different from the reported studies. Therefore, our study may be suitable in MP sensing.

Interference during the simultaneous detection of MP is caused by 2-nitrophenol, 3-nitrophenol, 4-nitrophenol, nitroaniline, KCl, Na_2_SO_4_, MgCl_2_, glucose, sucrose and CBR. These have been studied with the CP5-rGO-modified GCE, and the chemical structures of these interferences are shown at Fig. S12.† The currents for the reduction of 20 μM of MP at the CP5-rGO/GCE are compared with the signal obtained in the presence of a 50-fold higher concentration of interfering species. The presence of these interferents does not affect the MP responses (Fig. 4D). These results indicate that the system has better selectivity than other methods.

The CP5-rGO/GCE is used to measure the content of MP in soil and waste water samples employing standard addition methods to assess the feasibility of CP5-rGO-modified GCEs for real sample analysis. The procedure for preparing practical samples is elaborated as follows: soil (30 mg) was added to DW (150 mL) under sonication, the solution was then filtrated by filter membrane (0.45 μM) for removing insoluble solid matter. The waste water samples were collected from river water. The detection of MP in soil and waste water practical samples is carried out by standard addition methods. Table 1 shows the recoveries ranging from 98.6% to 101.2% and RSDs ranging from 2.1% to 4.3% for MP. The proposed method for cationic pillar[5]arenes-functionalized reduced graphene-modified GCE has satisfactory accuracy with potential applications in industrial and environmental sample analyses.

**Table 1:** Determination of MP in soil and tap water samples

Sample	Added (μM)	Measured (μM)	RSD (%)	Recovery (%)
Soil	0	—	—	—
	5	4.93 ± 0.07	2.1	98.60
	10	10.12 ± 0.14	3.9	101.2
	20	19.95 ± 0.18	3.1	99.75
Tap water	0	—	—	—
	5	4.97 ± 0.07	4.3	99.4
	10	9.97 ± 0.13	3.2	99.7
	20	20.23 ± 0.23	2.9	101.15

## Conclusion

Herein, we report a rapid and sensitive electrochemical sensor based on cationic pillar[5]arene functionalized reduced graphene-modified GCE CP5-rGO/GCE. These sensors were used to measure MP by DPV for the first time. Compared to β-CD-rGO/GCE, the proposed CP5-rGO/GCE sensor exhibits excellent electrochemical catalytic activity, rapid response, high sensitivity, good reproducibility and strong anti-interference ability. In addition, the described method can detect MP in soil and waste water samples with satisfactory results. A low detection limit of 0.0003 μM (S/N = 3) and a linear response range of 0.001–150 μM indicates that this is a promising electrochemical sensing platform that paves a promising path for facile, green and sensitive analysis of methyl parathion and other organophosphorus pesticides.

## Conflicts of interest

There are no conflict to declare.

## Supplementary Material

RA-009-C8RA08555B-s001
