# Supplementary material for: Ultrasensitive electrochemical detection of methyl parathion pesticide based on cationic water-soluble pillar[5]arene and reduced graphene nanocomposite
Source: RSC Adv. 2019 Jan 2;9(1):345–53. doi: 10.1039/c8ra08555b (PMC9059332; doi:10.1039/c8ra08555b)
Supplement: RA-009-C8RA08555B-s001 [file RA-009-C8RA08555B-s001.pdf]

Electronic Supplementary Material (ESI) for RSC Advances.
This journal is © The Royal Society of Chemistry 2018

Suppporting Information

Ultrasensitive electrochemical detection of methyl parathion pesticide based on cationic water-soluble pillar[5]arene and reduced graphene nanocomposite

Xiaoping Tan*, Yan Liu, Tingying Zhang, Shasha Luo, Xi Liu, Hexiang Tian, Yang Yang, Chunlian Chen

Key Lab of Inorganic Special Functional Materials, Chongqing Municipal Education Commission, School of Chemistry and Chemical Engineering, Yangtze Normal University, Chongqing, 408100, China

S1. Reagents and apparatus

Reagents and apparatus. CP5 was synthesized according to the previous papers procedures.[1,2] All reagents were commercially available of analytical grade and used as supplied without further purification. All aqueous solutions were prepared by the deionized water (DW, 18.25 MΩ cm). CHI 660E Electrochemical Workstation was purchased from Shanghai Chenhua Instrument (Shanghai, China). Cyclic voltammetry (CV) and differential pulse voltammetry (DPV) were performed via the three-electrode system, the modied GCE as working electrode, platinum wire as counter electrode, saturated calomel electrode (SCE) as reference electrode, respectively. NMR spectra was recorded on a Bruker AV. DRX5 instrument operated at 400 MHz and 500 MHz. Fourier transform infrared (FTIR) study was performed over the wavenumber, range of 4000–400 cm^{-1} by a Thermo Fisher SCIENTIFIC Nicolet IS10 (Massachusetts, USA) FTIR impact 410 spectrophotometer using KBr pellets. Thermogravimetric analysis (TGA) was carried out on a STA 449F3 TGA (TA Instruments, Selbu, Germany), from 25 to 800 °C in nitrogen at a heating rate of 10 °C min^{-1}. X-ray photoelectron spectroscopy (XPS) measurements were performed with Al Ka X-ray radiation as the X-ray source for excitation, which were carried out on an ESCALAB-MKII spectrometer (VG Co., United Kingdom). A Malvern Zetasizer Nano series was used for the zeta potential measurements.

S2. Synthesis of water-soluble cationic pillar[5]arene (CP5)

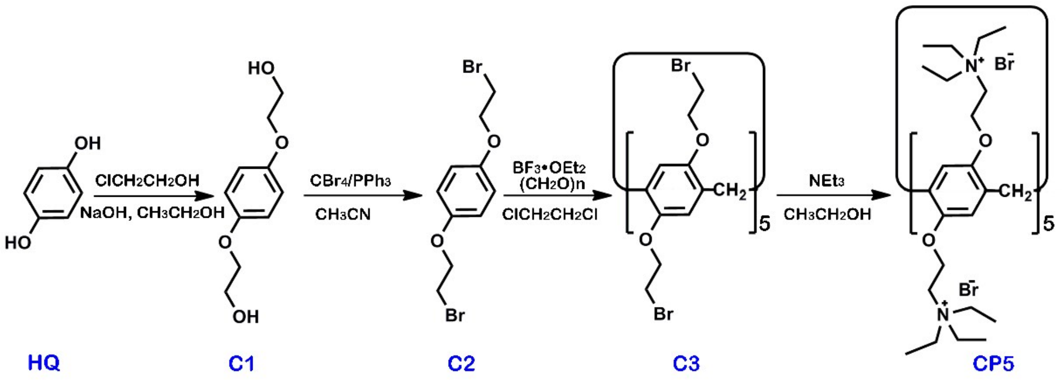

Scheme S1 Synthetic route of **CP5**.

Synthesis of C1: 30 mL of aqueous solution of NaOH (6.225 g, 155.6 mmol) was

added to 130 mL of ethanol solution containing 1,2-dihydroxybenzene (HQ, 5.715 g, 51.9 mmol) under stirring for 15 min followed by addition of 2-chloroethanol (12.55 g, 155.85 mmol) under refluxing for 72 h. After that, the pH of the mixture solution was adjusted to 6−7, and the solvent was removed by reduced pressure distillation. Ethanol (150 mL) was added to dissolve the residual solid at the elevated temperature, and then the hot mixture solution was filtered to remove insoluble inorganic salts. The filtrate was concentrated to get the crude product. The pure product **C1** was obtained by recrystallization in ethanol. The ^{1}H NMR spectrum of **C1** is shown in Figure S1. ^{1}H NMR (400 MHz, D_2O, rt) δ (ppm): 6.907 (s, 4H), 4.010 (t, J = 4 Hz, 4H), 3.803 (t, J = 4 Hz, 4H). The ^{13}C NMR spectrum of **C1** is shown in Figure S2. ^{13}C NMR (100 MHz, D_2O, rt) δ (ppm): 152.70, 116.23, 70.06, 60.13.

Synthesis of C2: A solution of **C1** (5.0 g, 25.2 mmol) and triphenylphosphine (16.0 g, 60 mmol) in dry acetonitrile (150 mL) was cooled with an ice bath. Under vigorous stirring, carbon tetrabromide (20.0 g, 60 mmol) was slowly added. The mixture was stirred at room temperature for 4 hours. Then cold water (150 mL) was added to the reaction mixture to give white precipitation. The precipitate was collected, washed with methanol/water (3:2, 3 × 100 mL), recrystallized from methanol, and dried under vacuum to afford **C2** as white crystals (7.2 g, 92%). The ^{1}H NMR spectrum of **C2** is shown in Figure S3. ^{1}H NMR (400 MHz, $CDCl_3$, rt) δ (ppm): 6.868 (s, 4H), 4.251 (t, J = 6.4 Hz, 4H), 3.621 (t, J = 6.4 Hz, 4H). The ^{13}C NMR spectrum of **C2** is shown in Figure S4. ^{13}C NMR (100 MHz, $CDCl_3$, rt) δ (ppm): 152.84, 116.10, 68.72, 29.32.

Synthesis of C3: Boron trifluoride diethyl etherate ($BF_3·OEt_2$, 3.26 g, 23.0 mmol) was added to the mixed solution of paraformaldehyde (0.349 g, 11.5 mmol) and **C2** (3.37 g, 11.5 mmol) in 1, 2-dichloroethane (100 mL) under nitrogen atmosphere. Then the mixture was stirred at room temperature for 1 hour. A green solution was got. The reaction mixture was then washed with water (2 × 100 mL) and dried with excess Na_2SO_4. After the solvent was removed, the obtained solid was purified by column chromatography on silica gel with petroleum ether/dichloromethane (1:2 *v*/*v*) as the eluent to get a white powder of **C3** (1.6 g, 41 %). The ^{1}H NMR spectrum of **C3** is shown in Figure S5. ^{1}H NMR (400 MHz, $CDCl_3$, rt) δ (ppm): 6.910 (s, 10H), 4.225 (t,

J = 5.2 Hz, 20H), 3.843 (s, 10H), 3.631 (t, *J* = 4.8 Hz, 20H). The ^{13}C NMR spectrum of **C3** is shown in Figure S6. ^{13}C NMR (100 MHz, $CDCl_3$, rt) δ (ppm): 149.91, 129.31, 116.35, 69.22, 30.92, 29.64.

Synthesis of CP5: An excess amount of triethyl amine (10.0 mL, 72 mmol) was added to a solution of **C3** (1.0 g, 0.595 mmol) in ethanol (50 mL), and the resulting mixture was refluxed for 48 hours. After cooling to room temperature, diethyl ether was added to the mixture and precipitate was obtained. The precipitate was filtered, and was washed by acetone and diethyl ether to remove excess triethyl amine and obtained the colorless solid of **CP5**. The ^{1}H NMR spectrum of **CP5** is shown in Figure S7. ^{1}H NMR (500 MHz, DMSO-*d6*, rt) δ (ppm): 7.019 (s, 10H), 4.529 (br, 20H), 4.03 (br, 20H), 3.766 (s, 10H), 3.523 (br, 60 H), 1.264 (t, *J* = 6 Hz, 90H). The ^{13}C NMR spectrum of **CP5** is shown in Figure S8. ^{13}C NMR (100 MHz, D_2O, rt) δ (ppm): 149.15, 130.69, 116.68, 63.19, 54.41, 53.59, 29.60, 6.99.

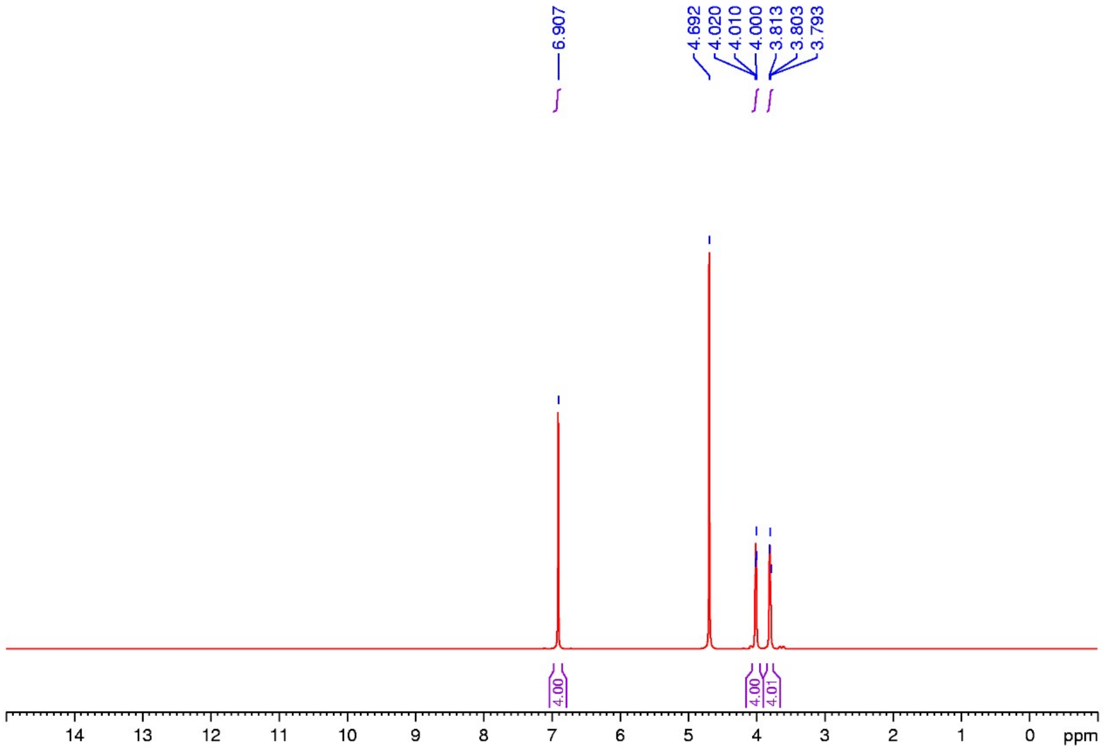

Fig. S1 ^{1}H NMR spectrum (400 MHz, D_2O, 298 K) of **C1**.

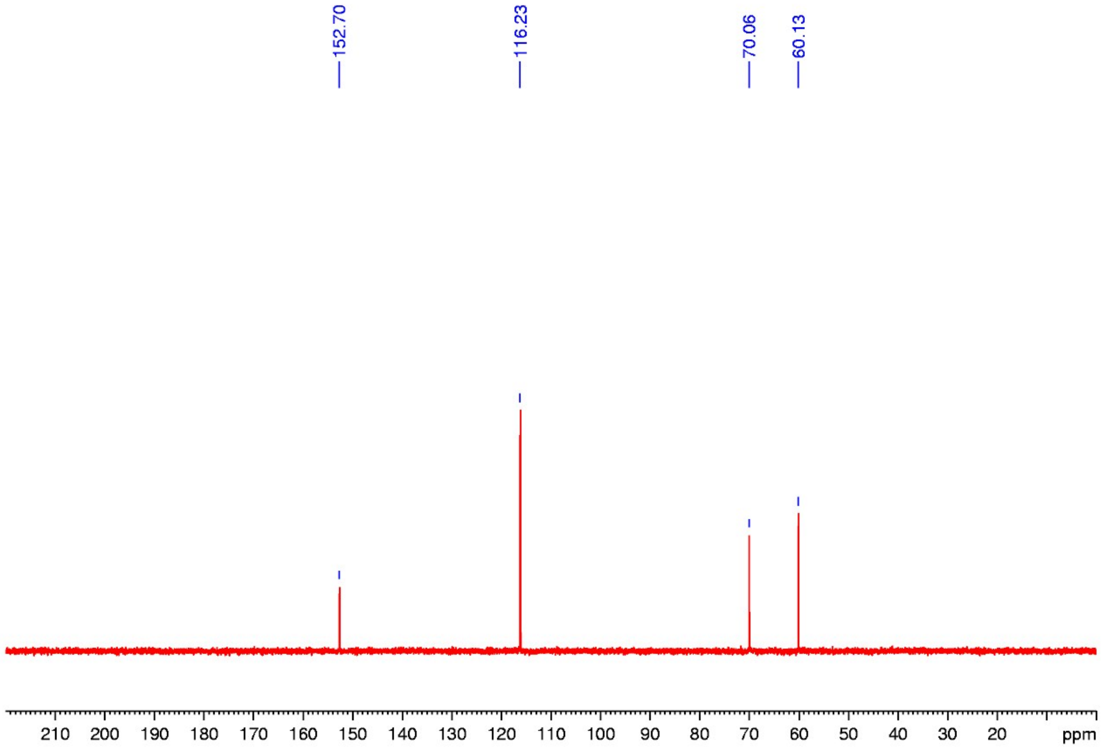

Fig. S2 ^{13}C NMR spectrum (100 MHz, D_2O, 298 K) of **C1**.

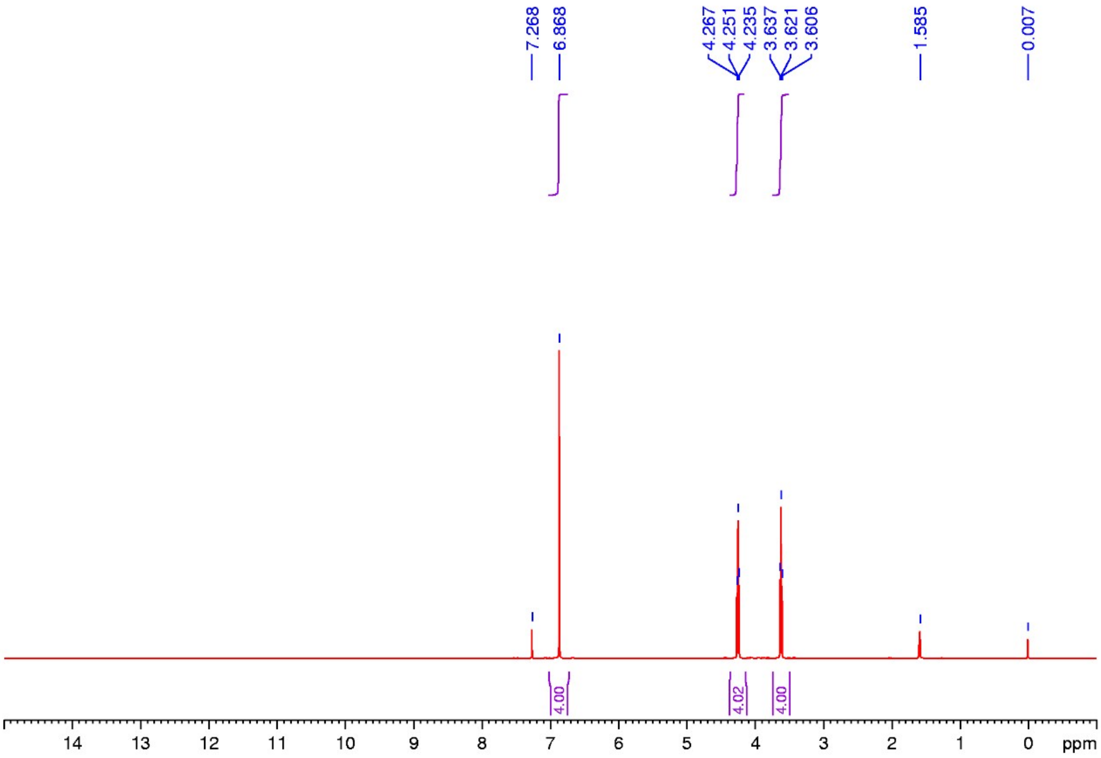

Fig. S3 ^{1}H NMR spectrum (400 MHz, $CDCl_3$, 298 K) of **C2**.

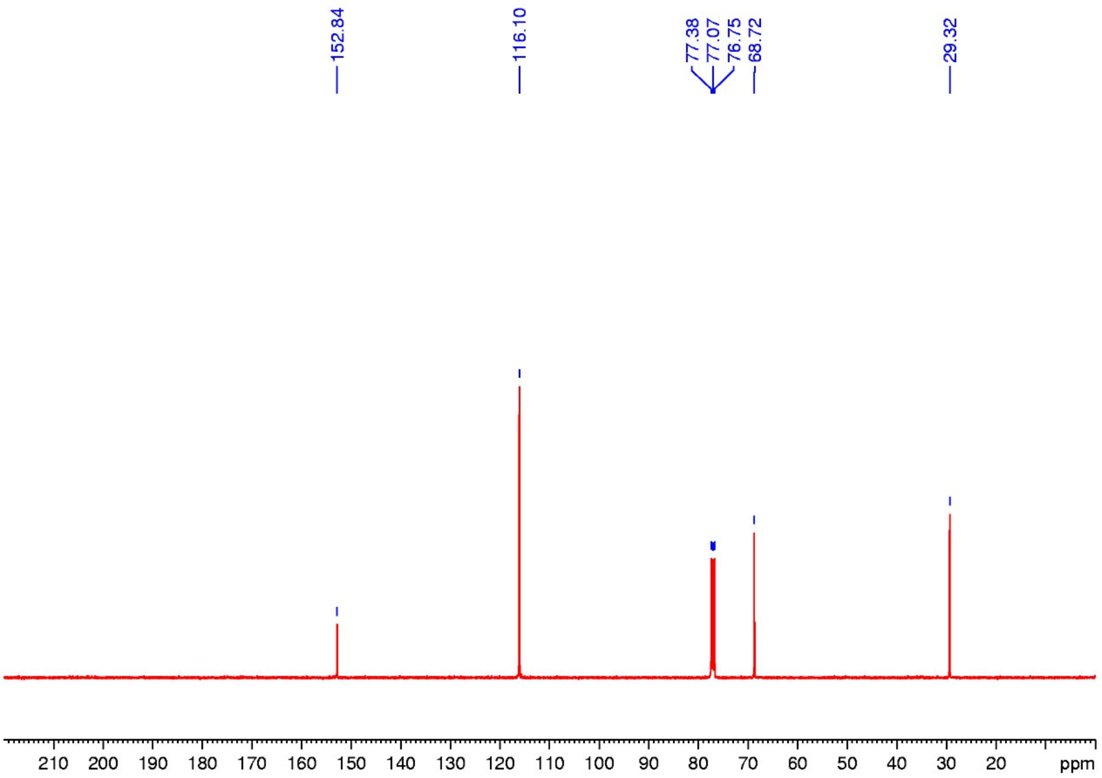

Fig. S4 ^{13}C NMR spectrum (100 MHz, $CDCl_3$, 298 K) of **C2**.

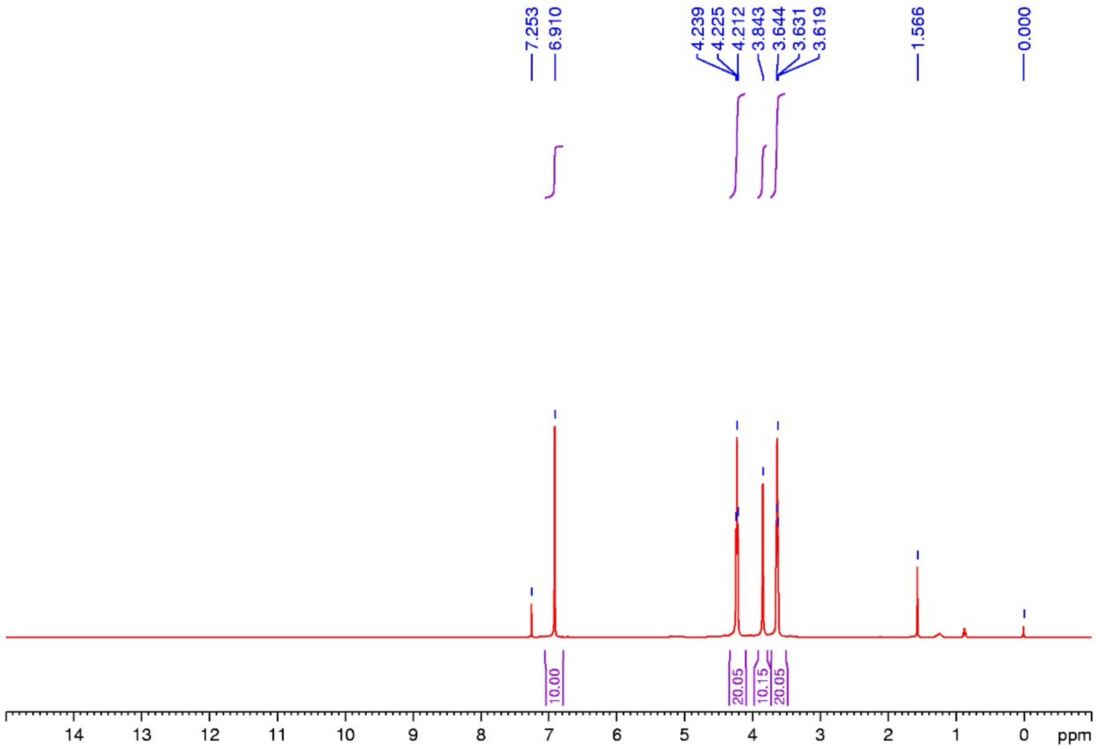

Fig. S5 ¹H NMR spectrum (400 MHz, $CDCl_3$, 298 K) of **C3**.

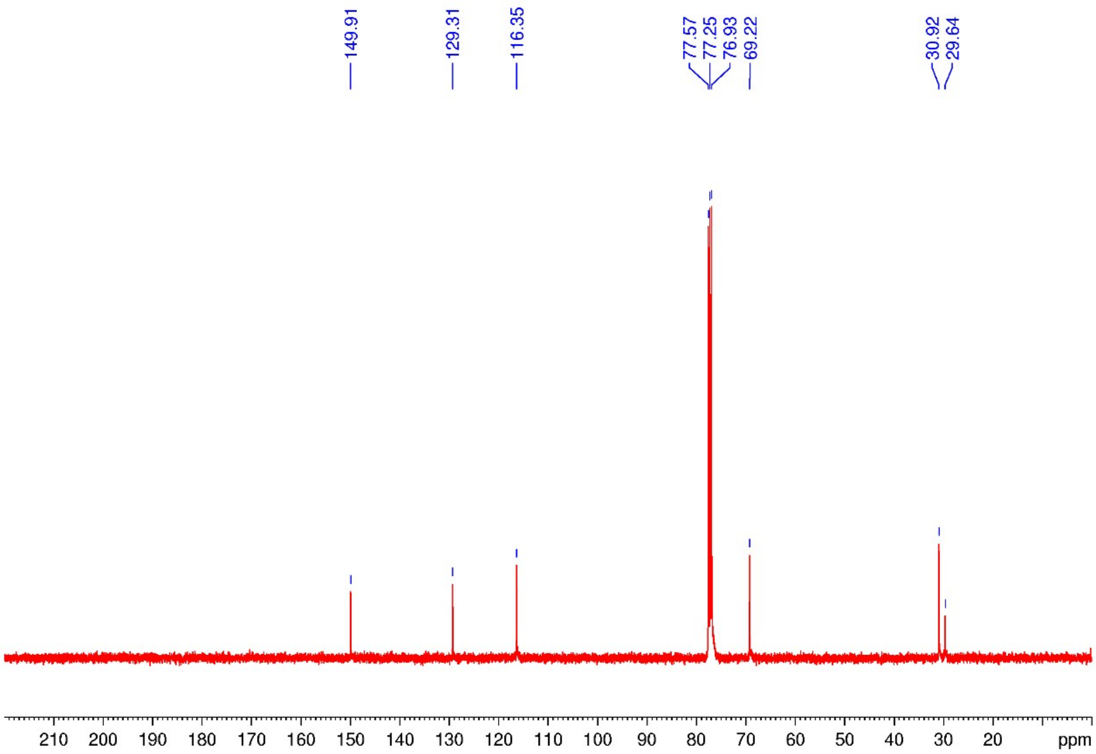

Fig. S6 ¹³C NMR spectrum (100 MHz, $CDCl_3$, 298 K) of **C3**.

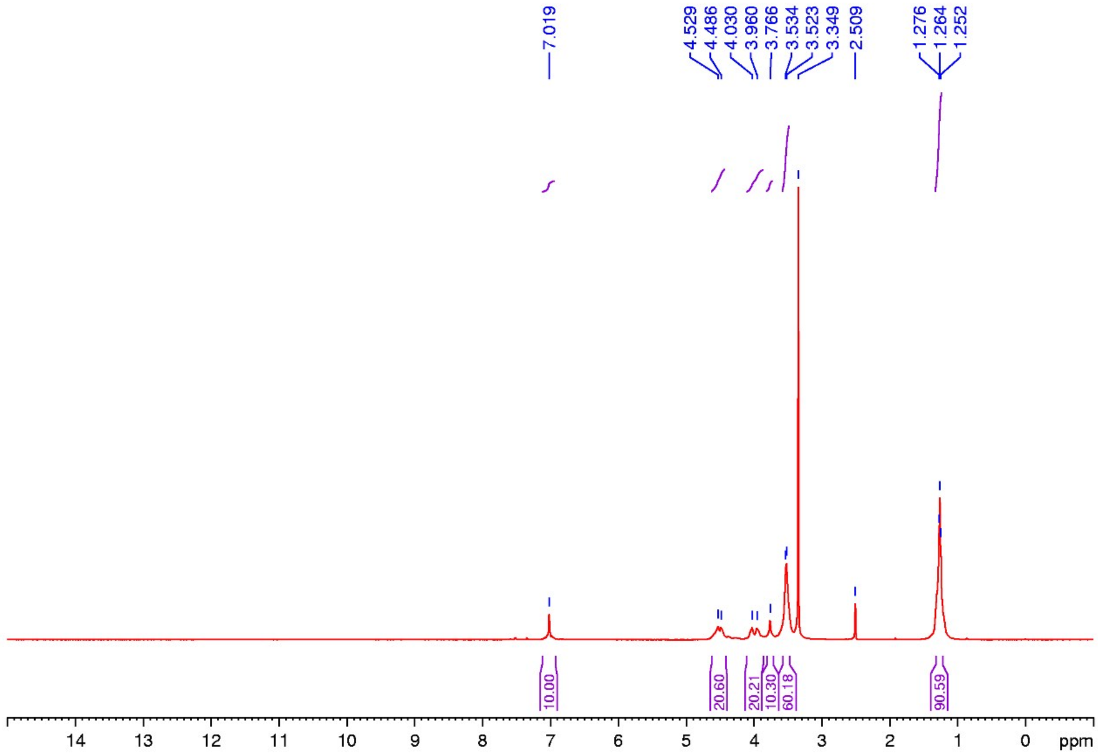

Fig. S7 ¹H NMR spectrum (500 MHz, DMSO-*d6*, 298 K) of **CP5**.

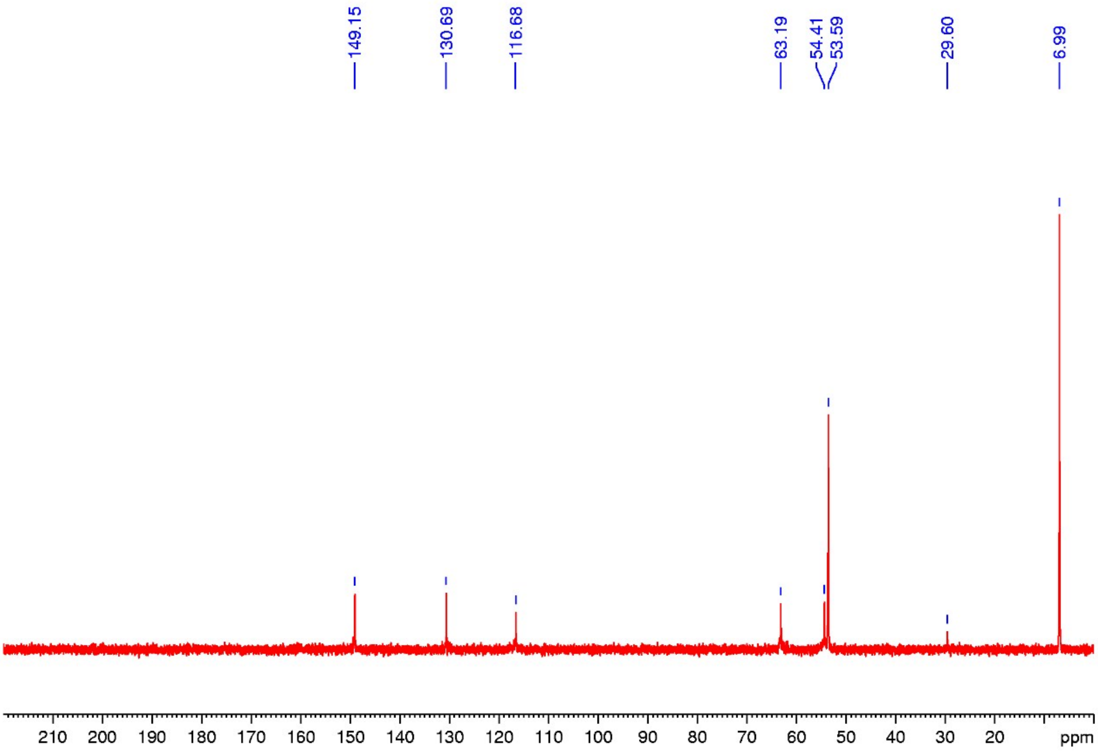

Fig. S8 ^{13}C NMR spectrum (100 MHz, D_2O, 298 K) of **CP5**.

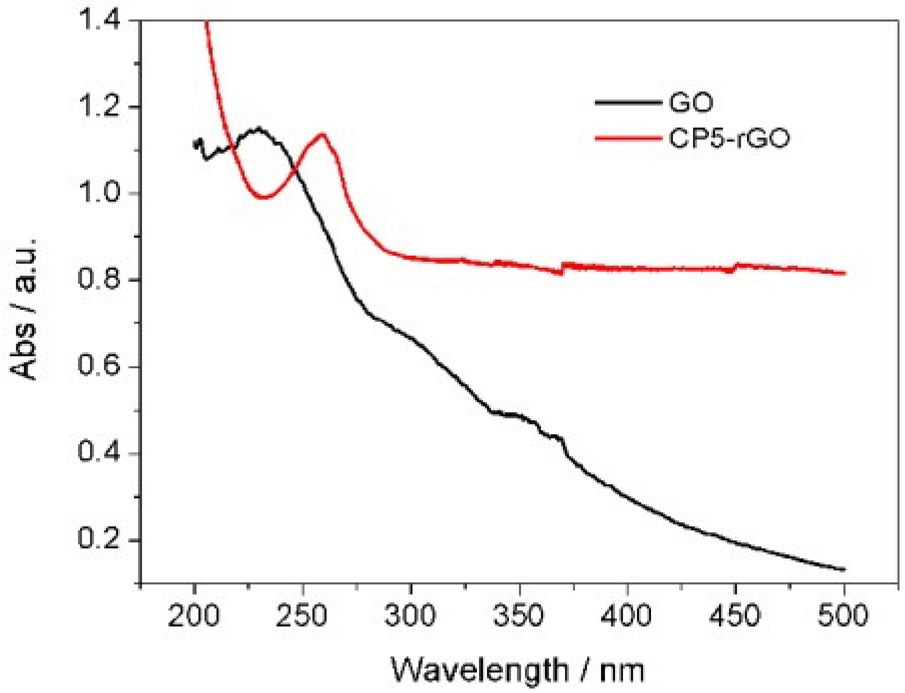

Figure S9. UV/vis spectra of the GO and CP5-rGO in aqueous solution.

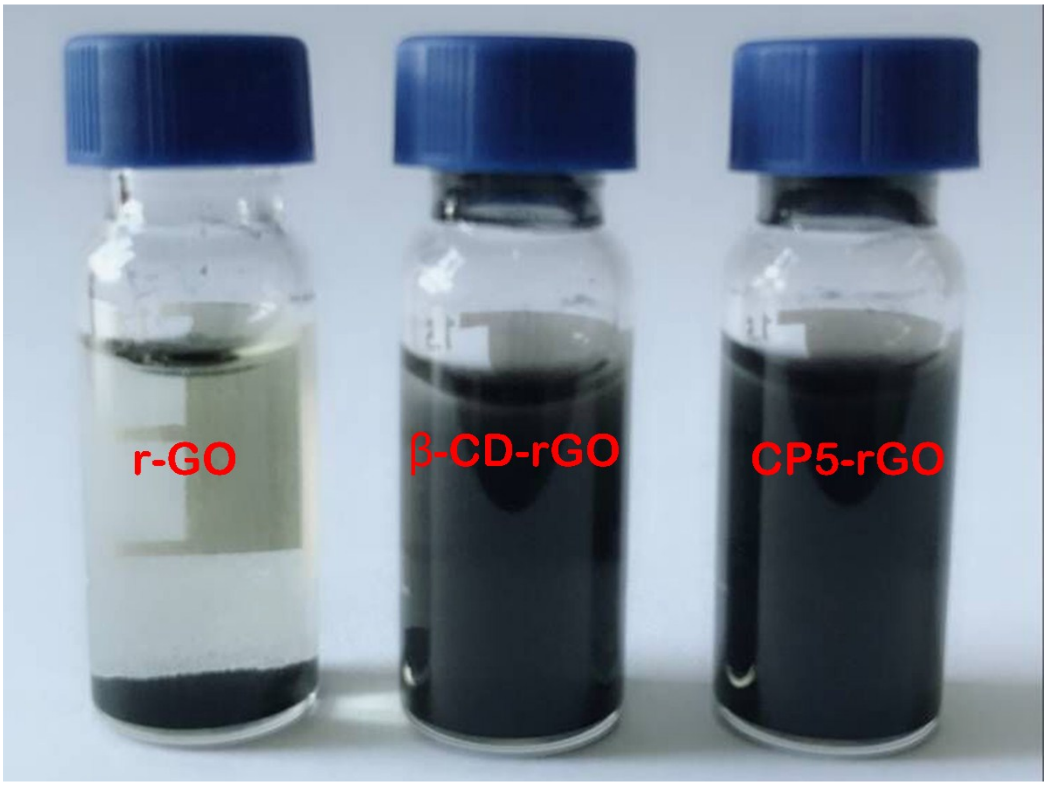

Fig. S10 Photograph of r-GO, β-CD-rGO, and CP5-rGO aqueous dispersions.

.

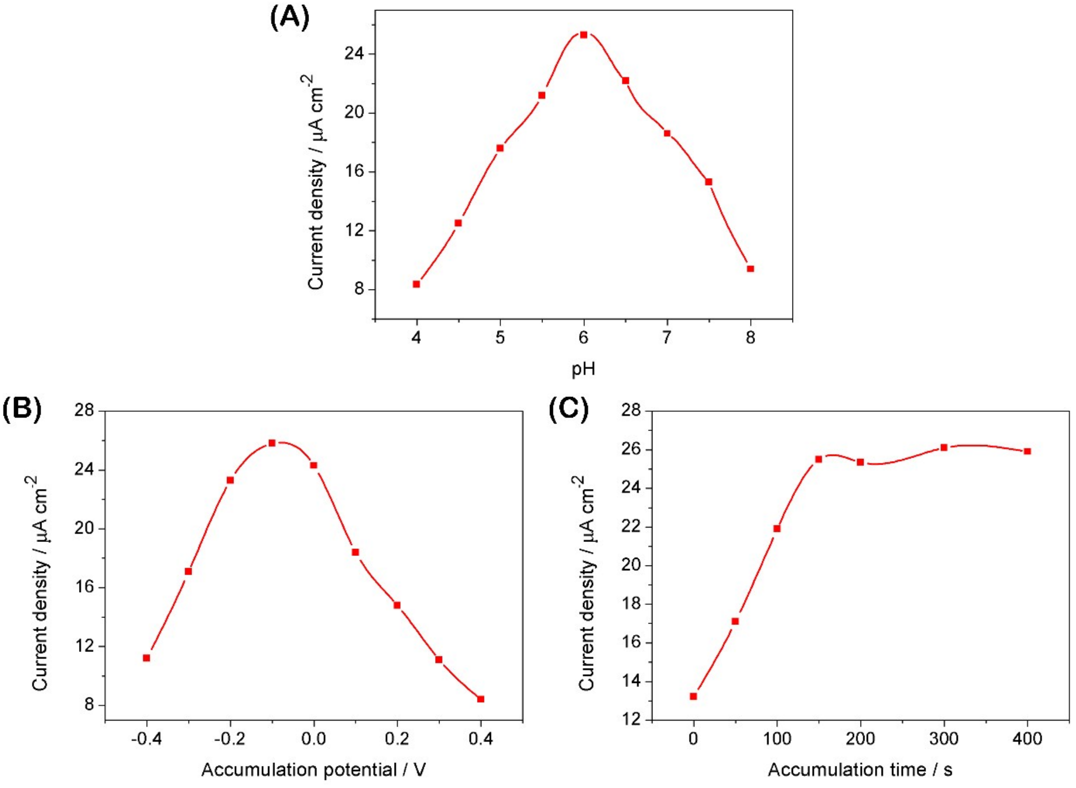

Fig. S11 The effect of the pH (A), the accumulation potential (B), and the accumulation time (C) on the DPV peak currents of 10 μM MP at the CP5-rGO modified electrode in 0.1 M PBS.

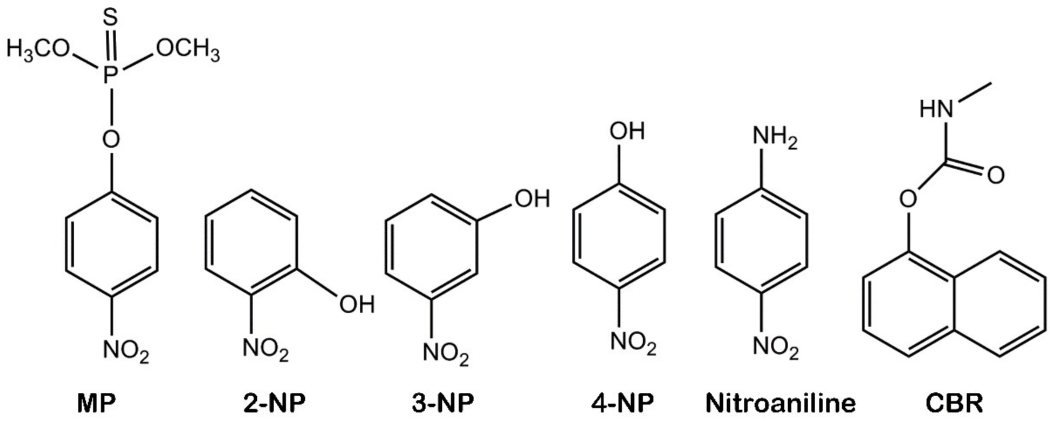

Fig. S12 The chemical structures of MP, 2-NP, 3-NP, 4-NP, nitrianiline and CBR, respectively.

Table S1 Comparison of different electrode for quantitative detection of MP

Modified electrode	Method	Liner range (μM)	LOD (μM)	Ref
Au/Nafion/GCE	DPV	0.5–120	0.1	3
Silicate-CTAB/GCE	DPV	0.1-100	0.01	4
MWCNTs-Chitosan/GCE	DPV	0.19-7.6	0.019	5
MWCNTs-PAAM/GCE	DPV	0.005-10	0.002	6
GN-AuNRs/GCE	SWV	0.04-1.9<br>2.85-15.2	0.003	7
SH-β-CD/AuNPs/SWCNTs/GCE	SWV	0.002-0.08	0.0001	8
AChE/Au-PPy/GCE	DPV	0.02-0.46<br>1.90-17.1	0.01	9
N-GS/GCE	CV	0.38-38	0.04	10
CP5-rGO/GCE	DPV	0.001-150	0.0003	This work

Reference

1 R. Joseph, A. Naugolny, M. Feldman, I. M. Herzog, M. Fridman, Y. Cohen, , J. Am. Chem. Soc., 2016, **138**, 754.

2 Y. J. Ma, X. F. Ji, F. Xiang, X. D. Chi, C. Y. Han, J. M. He, Z. Abliz, W. X. Chen, F. H. Huang, Chem. Commun., 2011, **47**, 12340.

3 T. F. Kang, F. Wang, L.P. Lu, Y. Zhang, T.S. Liu, Sens. Actuators, B: Chem., 2010, 145, 104.

4 S. F. Xia, J. F. Zhang, C.Y. Li, Anal. Bioanal. Chem., 2010, **396**, 697.

5 D. Du, M. H. Wang, J. M. Zhang, J. Cai, H. Y. Tu, A. D. Zhang, Electrochem. Commun., 2008, **10**, 85–89.

6 Y. B. Zeng, D. Yu, Y. Y. Yu, T. S. Zhou, G. Y. Shi, J. Hazard. Mater. 2012, **217**, 315.

7 W. X. Zhu, W. Liu, T. B. Li, X. Y Yue, T. Liu, W.T . Zhang, S. X. Yu, D. H. Zhang, J. L. Wang, Electrochim. Acta, 2014, **146**, 419.

8 X. C. Fu, J. Zhang, YY. Tao, J. Wu, CG. Xie, L. T. Kong, Electrochim. Acta, 2015, **153**, 12.

[9] J. Gong, L. Wang, L. Zhang, Biosens. Bioelectron., 2009, **24**, 2285.

[10] X. D. Xue, Q. Wei, D. Wu, H. Li, Y. Zhang, R. Feng, B. Du, Electrochim. Acta, 2014, **116**, 366.
